# Identifying patients at high risk for antibiotic treatment following hospital admission: a predictive score to improve antimicrobial stewardship measures

**DOI:** 10.1007/s15010-025-02525-9

**Published:** 2025-04-15

**Authors:** Moritz Beck, Carolin Koll, Uga Dumpis, Christian G. Giske, Siri Göpel, Silje Bakken Jørgensen, Johanna Kessel, Lars Kaare Kleppe, Dorthea Hagen Oma, Noa Eliakim Raz, Makeda Semret, Gunnar Skov Simonsen, Maria J. G. T. Vehreschild, Kerstin Albus, Lena M. Biehl, Jörg J. Vehreschild, Annika Y. Classen, the PILGRIM study group, Lena M. Biehl, Pauls Aldins, Per Espen Akselsen, Anne Mette Asfeldt, Nadine Conzelmann, Kelly Davison, Thilo Dietz, Simone Eisenbeis, Lucas J. Fein, Fe dja Farowski, Romina Georghe, Maayan Huberman Samuel, Barbara Ann Jardin, Merve Kaya, Christian Kjellander, Zane Linde Ozola, Leonard Leibovici, Nick Schulze, Hannes Wåhlin, Aija Vilde, Viesturs Zvirbulis

**Affiliations:** 1 Department I of Internal Medicine, Division of Infectious Diseases, Faculty of Medicine and University Hospital of Cologne, University of Cologne, Kerpener Str. 62, 50937 Cologne, Germany; 2 Department of Urology, Hospital of Leverkusen, Leverkusen, Germany; 3 Faculty of Medicine, University of Latvia, Riga, Latvia; 4 Pauls Stradins Clinical University Hospital, Riga, Latvia; 5 Department of Laboratory Medicine, Karolinska Institute, Stockholm, Sweden; 6 Department of Clinical Microbiology, Karolinska University Hospital, Stockholm, Sweden; 7 Department of Internal Medicine I, University Hospital Tübingen, Tübingen, Germany; 8 German Centre for Infection Research (DZIF), Clinical Research Unit for Healthcare Associated Infections, Tübingen, Germany; 9 Department of Medical Microbiology and Infection Control and Department of Emergency Care, Akershus University Hospital, Lørenskog, Norway; 10 Department II of Internal Medicine, Infectious Diseases, University Hospital Frankfurt, Goethe University Frankfurt, Frankfurt Am Main, Germany; 11 Department of Infection Prevention and Control, Stavanger University Hospital, Stavanger, Norway; 12 Section for Patient Safety, Haukeland University Hospital, Bergen, Norway; 13 Internal medicine E, Rabin medical center Beilinson campus, Petah-Tikva, Israel; 14 Faculty of Medicine, Tel Aviv University, Tel Aviv, Israel; 15 Infectious Diseases and Medical Microbiology, Mcgill University Health Centre, Montreal, Canada; 16 Department of Microbiology and Infection Control, University Hospital of North Norway, Tromsø, Norway; 17 Uit the Arctic University of Norway, Tromsø, Norway; 18 German Centre for Infection Research, Partner Site Bonn-Cologne, Cologne, Germany; 19 Faculty of Medicine and University Hospital Cologne, Institute of Translational Research, Cologne Excellence Cluster on Cellular Stress Responses in Aging-Associated Diseases (CECAD), University of Cologne, Cologne, Germany; 20 Faculty of Medicine and University Hospital of Frankfurt, Institute for Digital Medicine and Clinical Data Sciences, Goethe University Frankfurt, Frankfurt am Main, Germany

**Keywords:** Antimicrobial stewardship, Antibiotic treatment, Prediction score, Clinical trial

## Abstract

**Purpose:**

Identifying patients for clinical studies evaluating strategies to reduce unnecessary antibiotic usage in hospitals is challenging. This study aimed to develop a predictive score to identify newly hospitalized patients with high likelihood of receiving antibiotics, thus improving patient inclusion in future studies focusing on antimicrobial stewardship (AMS) programs.

**Methods:**

This retrospective analysis used data from the PILGRIM study (NCT03765528), which included 1,600 patients across ten international sites. Predictive variables for antibiotic treatment during hospitalization were computed, and an additive score model was developed using logistic regression and 10-fold cross-validation. The PILGRIM score was validated in an independent cohort (validation cohort), with performance metrics assessed.

**Results:**

Data from 1,258 patients was included. In the development cohort 52.8% (n = 445) and in the validation cohort 42.4% (n = 134) of patients received antibiotics. Key predictors included hematologic malignancies, immunosuppressive medication, and past hospitalization. The logistic regression model demonstrated an area under the curve of 0.74 in the validation. The final additive score incorporated these predictors plus “planned elective surgery” achieving a specificity of 92%, a positive predictive value of 78%, a sensitivity of 41%, and a negative predictive value (NPV) of 69%in validation set.

**Conclusion:**

The PILGRIM score effectively identifies newly hospitalized patients likely to receive antibiotics, demonstrating high specificity and PPV. Its application can improve future AMS programs and trial recruitment by facilitating targeted inclusion of patients, especially in the hematological and oncological setting. Further -external and prospective- validation is needed to broaden the model’s applicability.

**Supplementary Information:**

The online version contains supplementary material available at 10.1007/s15010-025-02525-9.

## Introduction

The World Health Organization (WHO) has named antimicrobial resistance (AMR) as one of the most relevant global health threats of the current century. According to recent estimates, AMR contributed to approximately 5 million deaths globally in 2019, 1.3 million of which were directly attributable to infections by resistant pathogens [1]. AMR complicates the treatment of bacterial infections, leading to increased mortality and morbidity, especially in high-risk patient cohorts [2, 3]. Inappropriate and excessive use of antibiotics in both human and veterinary medicine is one of the key drivers behind the rapid spread of resistant organisms. Its effects are exacerbated by dense human and animal populations, increased international trade, travel and migration [4, 5].

Antimicrobial stewardship (AMS) programs aim to optimize antibiotic use by promoting appropriate prescription behavior and reducing unnecessary antibiotic exposure through sets of interventions, and thereby improving patient outcomes [6]. Effective AMS interventions can lead to shortened duration of antibiotic treatment and hospitalizations, and improved adherence to clinical treatment guidelines [7]. However, these interventions are most impactful when applied to patient populations with high likelihood of receiving antibiotics. Identifying such patients early in their hospital stay or even upon admission is crucial for the targeted inclusion in clinical trials evaluating AMS strategies but also to improve implementation of AMS programs.

Despite the importance of identifying high-risk patient populations, there is a lack of robust, validated predictive models that can accurately forecast the need for antibiotic treatment in newly hospitalized patients. To our knowledge, existing studies on risk factors for future antibiotic use are very limited, with so far only one study describing a logistic regression model to assess the risk of subsequent antibiotic treatment in newly admitted patients [8].

This study aims to fill this gap through the development of an easy-to-use predictive scoring model, the PILGRIM score, that helps to identify patients at high risk of receiving antibiotic treatment early during hospitalization. By integrating patient-level risk factors into a scoring system, the PILGRIM score can facilitate patient stratification for AMS interventions and clinical trials leading to reductions in inappropriate antibiotic use and thereby improving outcomes.

## Methods

### Data collection: the PILGRIM study

This analysis is based on data from the PILGRIM study (Impact of Prescription Quality, Infection Control and Antimicrobial Stewardship on Gut Microbiota Domination by Healthcare-Associated Pathogens; ClinicalTrials.gov identifier: NCT03765528), a multicenter, international, prospective cohort study conducted from 2019 to 2022. The PILGRIM study aimed to examine the effects of antibiotic treatment on the gut microbiota, with a focus on its role in the development of colonization and domination of the gut by healthcare-associated pathogens. The study involved ten sites located in six countries: Germany, Sweden, Latvia, Norway, Israel, and Canada. Patients included in the study were those admitted to the hospital with a planned or suspected high likelihood of receiving systemic antibiotic treatment for a duration of ≥ 5 days within the first 10 days of their stay, but without recent antibacterial exposure (≤ 14 days) except for prophylaxis with trimethoprim/sulfamethoxazole. Additionally, patients who have received courses of systemic antibacterials for 7 days or more within the past two months were excluded. Detailed inclusion and exclusion criteria can be found in the supplementary material (see Supplementary Table 1). The likelihood of antibiotic exposure was estimated based on the recruiting physician’s perspective on risk factors (e.g. comorbidities) and admission reason (e.g. chemotherapy). Moreover, recruitment focused on departments with typical high rates of antibiotic treatments (e.g. hematology and oncology). Upon initiation of this sub-analysis a total of 1600 patients had been recruited. Clinical data were collected for each patient in a standardized electronic case report form (eCRF) and longitudinal stool sample collection was performed during the study. If an antibiotic treatment was initiated during the study period, an in-depth data documentation and sample collection was triggered. The clinical dataset included a wide range of variables, including patient demographics, comorbidities, medication use and preceding hospitalizations. Results of the sample analysis were not used for this sub-analysis.

The PILGRIM study was approved by the ethics committees of all participating sites (ID of lead committee in Cologne: UKK 18–316) and written informed consent of all participants was obtained prior to any study related measure. The study was conducted in accordance with the Declaration of Helsinki.

### Data preparation

Data analysis and score construction was performed with the *Statistical Package for Social Sciences* (SPSS®) of the International Business Machine Corporation (IBM Inc.®) Version 28.0.0.0. The dataset including 1600 patient cases was carefully monitored and cleaned to remove implausible or incomplete entries. Missing values were coded appropriately, and false entries (e.g., incorrect birth year, height, or weight) were queried or excluded. Variables with consistently low response rates or below a predefined threshold (e.g., rare comorbidities) were excluded from the analysis. The final set used for analysis comprised 41 different variables (see Supplementary Table 4). In total, following data cleaning 1,258 cases were included into the presented analysis.

To facilitate statistical analysis, key variables were recoded, grouped or aggregated (i.e. patient age groups, BMI from available height and weight data). Comorbidities were quantified using the Charlson Comorbidity Index (CCI), a widely used tool for assessing the burden of chronic illness [9]. Additionally, patient diagnoses and comorbidities were coded according to the International Classification of Diseases (ICD- 10), and groups of related diagnoses were aggregated to simplify the analysis.

The primary endpoint was the initiation of antibiotic treatment during hospitalization. Perioperative antibiotic prophylaxis was not counted as antibiotic treatment.

The dataset was non-randomly split in two sets: a development-dataset, containing 75% (n = 942) of all cases, beginning with those patients recruited first and a validation-dataset, containing the last recruited 25% (n= 316) patients. The validation set was not analyzed until final validation of the PILGRIM score, that was elaborated with the development-dataset.

In terms of descriptive statistics, for applicable variables (i.e. patient demographics, department responsible for inpatient stay) measures of central tendency (mean, median) and variability (standard deviation, interquartile range) were calculated. These were calculated separately per dataset, and both were compared to detect potential imbalances.

### Score development

**Fig 1:**
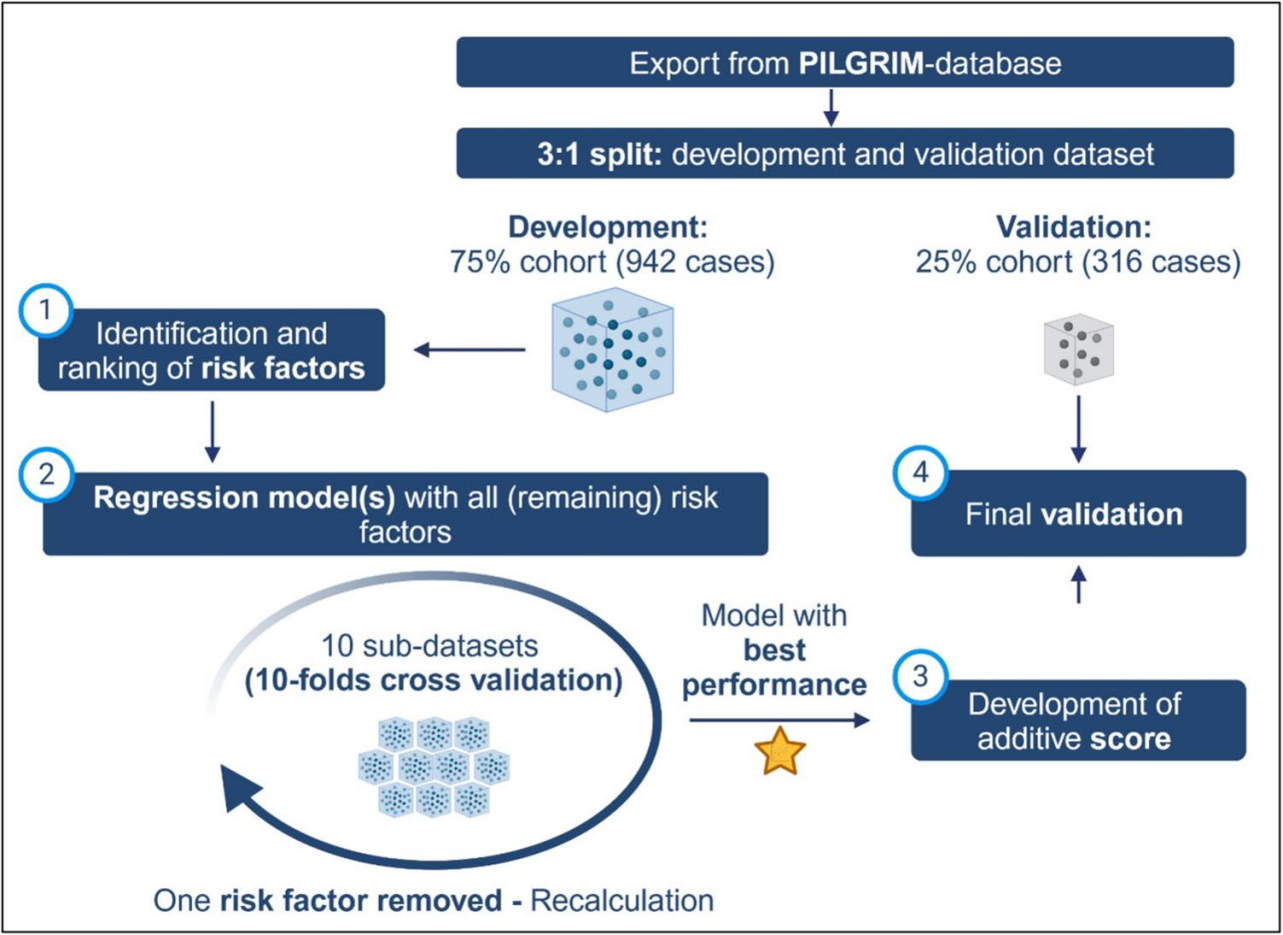
Workflow of the explorative data analysis: Starting with the 3:1 non-random split of the available PILGRIM dataset, the score-development was continued with the development dataset by (1) identification and ranking of risk factors, (2) calculation of multiple regression models with identified risk factors in an iterative manner, (3) development of an additive score model based on the regression model with the best performance and (4) final score validation based on the formerly separated validation dataset, Created in https://BioRender.com

The overarching steps of the explorative data analysis are shown in Figure 1 and described in more detail below.

First, associations between all available and applicable variables and the endpoint were analyzed (see Figure 1, step 1). Crosstabulations and Pearson’s chi-square (or Fisher’s exact test) were used to calculate respective p-values as well as Phi ɸ or Cramérs V for bigger tables to assess the power of the association. Simultaneously using multiple univariate regression models for all variables, regression-coefficients, p-values, odds ratios (OR) and respective 95%-confidence-intervals (95% CI) were calculated. Potential predictors were ranked based on their statistical significance (according to results of Pearson’s chi-square test).

**Fig 2:**
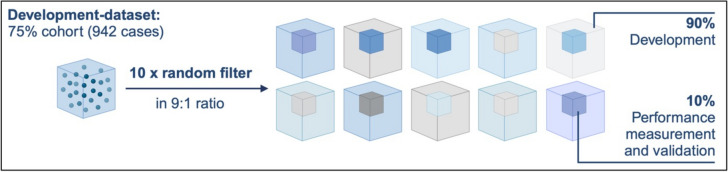
Illustration of the 10-fold cross-validation used for the logistic regression model described in step 2 of the methods section: based on the development dataset 10 filters were programmed, each separating the dataset in the ratio 9:1. The dataset size always remained the same. In each iteration of the regression models, the models were calculated 10-fold, each fold with different patients for the 90%-development and 10%-validation part, Created in https://BioRender.com

Subsequently, logistic regression models were performed to develop a prediction model identifying patients with antibiotic treatment (see Figure 1, step 2): to ensure robustness, a 10-fold cross-validation approach was applied, where the development dataset was randomly partitioned into 10 subsets. Each subset was used as a validation set once, while the remaining nine subsets were used to train the model. This process ensured that every case contributed to both model training and validation (see Figure 2).

**Fig 3:**
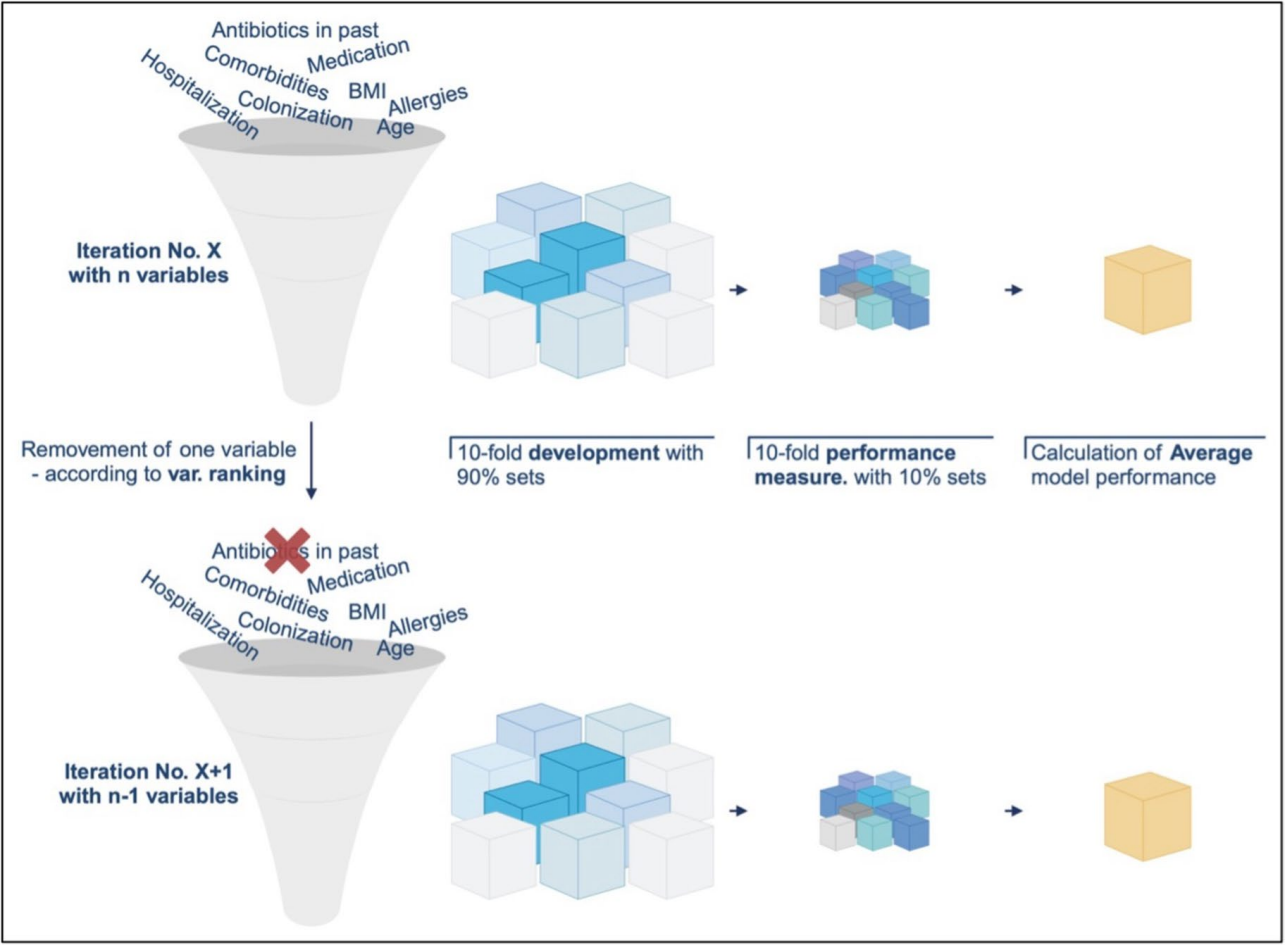
Process of iterative logistic regression model calculations incl. 10-folds cross validation: in the first iteration, all available variables were included and using 10-folds cross validation an average model performance was calculated. In the following iteration the least significant variable was removed (n- 1) from the model and the model was calculated again and so forth, Created in https://BioRender.com

The calculation of regression models itself was guided by an iterative process of logistic regression modeling. The above-mentioned ranking of variables informed the subsequent development of multivariate logistic regression models, which aimed to identify a subset of variables that could accurately predict the risk for initiation of an antibiotic treatment. Following standard procedures for model development, the initial logistic regression model included all identified predictors. In each subsequent iteration, the least significant variable (p-value) was removed, and the model was recalculated using the previously explained 10-fold cross-validation (see Figure 3) until only one predictor was left in the model.

Model performance for every iteration was assessed using several key metrics: sensitivity, specificity, positive predictive value (PPV), negative predictive value (NPV), and the area under the receiver operating characteristic curve (AUC) calculated as means from 10-folds cross-validation (see Figure 3). The selection process of the final logistic regression model started with the last model including the fewest variables before the performance of the models dropped due to missing significant predictive variables. Based on this, identified model variables were added and removed manually to identify the best combination based on its ability to balance sensitivity and specificity, while maintaining high overall predictive accuracy with the lowest number of variables possible.

Third, the score construction took place (see Figure 1, step 3). The PILGRIM score was derived from the final multivariate logistic regression model. Each predictor included was assigned a weight based on its regression coefficient, with higher weights assigned to stronger predictors. The score was then calculated as the sum of these weighted predictors. Three different methods for weighting were considered based on the calculations of the final regression model:

1. Werfel et al. [10]—Weights based on the ratio of each variable’s regression coefficient to the smallest regression coefficient in the model.
2. Matsushita et al. [11]—Weights based on the average of the two smallest regression coefficients.
3. Schneeweiss et al. [12]—Weights based on the natural logarithm of the odds ratios (ORs) associated with each predictor.

For comparison, an unweighted score (each predictor was assigned a weight of 1) was calculated. The performance of the weighted and unweighted scores was compared using sensitivity, specificity, PPV, NPV, and AUC metrics.

In a final step, the score was validated (see Figure 1, step 4) using the validation dataset. The same metrics used in model development were calculated for this dataset to assess the generalizability and stability of the score. The score performance was evaluated across a range of cut-off values, with the goal of identifying a threshold that maximized sensitivity and specificity.

## Results

### Descriptive statistics

In the development dataset, 942 patients were included, of which 445 patients (52.8%) received antibiotic treatment during their hospitalization (see Table 1). The cohort consisted of 580 men (61.6%), the median age of patients was 64 years (range: 18–91 years) and the mean body mass index (BMI) was 27.3 kg/m^2^. A significant portion of the cohort (35.9%, n = 338) had a CCI score of ≥ 5, indicating a high burden of chronic illness. About 39% (n = 367) of patients have been hospitalized in the six months prior to study inclusion, and 15% (n = 141) have been treated with antibiotics during this period. Notably, 44.5% (n = 419) of patients had a planned elective surgery during their hospital stay, while one-third (33.3%, n = 314) were receiving immunosuppressive medication either at the time of study enrollment or within the previous four weeks. When split by endpoint there were significant differences for the distribution of sex, age, past hospitalizations and antibiotic treatments, planned elective surgeries and immunosuppressive drugs (see Table 1).

**Table 1:** Development cohort: basic demographics of patients included with and without antibiotic treatment

	Endpoint positive patients (= received antibiotics) N = 445	Endpoint negative patients (= did not receive antibiotics) N = 497	Correlations (p-value)
Female sex	35.0% (n= 156)	41.4% (n = 206)	**0.044**
Age (median/IQR)	62.0 a/15.5	66.0 a/17.0	**0.048**
BMI (median/IQR)	26.8 kg/m^2^/6.1	26.5 kg/m^2^/6.8	0.441
CCI (median/IQR)	4.0/3.0	4.0/3.0	0.169
Past Hospitalization (last 6 months)	48.8% yes (n = 217)	31.0% yes (n = 154)	**< 0.001**
Antibiotics in the past (last 6 months)	20.9% yes (n= 93)	9.5% yes (n = 47)	**< 0.001**
Planned elective surgery	38.9% yes (n = 173)	49.5% yes (n = 246)	**< 0.001**
Immunosuppressive drugs	46.1% yes (n = 205)	21.9% yes (n = 109)	**< 0.001**

*p* < 0.05 = significant

*BMI* body mass index, *CCI* Charlson comorbidity index

In terms of hospital departments, the largest proportion of patients (39.4%, n = 371) was admitted to hematological and oncological wards. Other major departments were cardiothoracic surgery (25%, n = 236), cardiology (6.7%, n = 63), and general mainly abdominal surgery (5.8%, n = 54). Patients admitted to the hematology and oncology wards tended to be younger (median age 60 years) compared to the overall cohort (see Table 2).

**Table 2:** Development cohort: admitting departments and performed surgeries

Admitting departments	Proportions [%]
Hematological and oncological	39.4
Cardiothoracic surgery	25.0
Cardiology	6.7
General surgery	5.8
(General) internal medicine	5.2
Other	17.2

Type of elective surgery (applicable for patients with planned elective surgery)	
Cardiac surgery	56.0
Abdominal surgery	19.0
Thoracic surgery	5.0
ENT	5.0
Vascular surgery	4.0
Other/missing	10.0

*ENT* ear nose throat surgery

The validation dataset comprised 316 patients, with a similar distribution of patient characteristics to the development dataset (see Supplementary Table 2). Of these, 134 patients (42.4%) received antibiotics, while 182 patients (57.6%) did not. The percentage of patients admitted to hematology and oncology units (30.7%) or cardiothoracic surgery (18.7%) was lower than in the development dataset. The distribution of performed surgeries showed similar results although with smaller cardiothoracic proportion (see Supplementary Table 3).

### Risk factor identification

**Fig 4:**
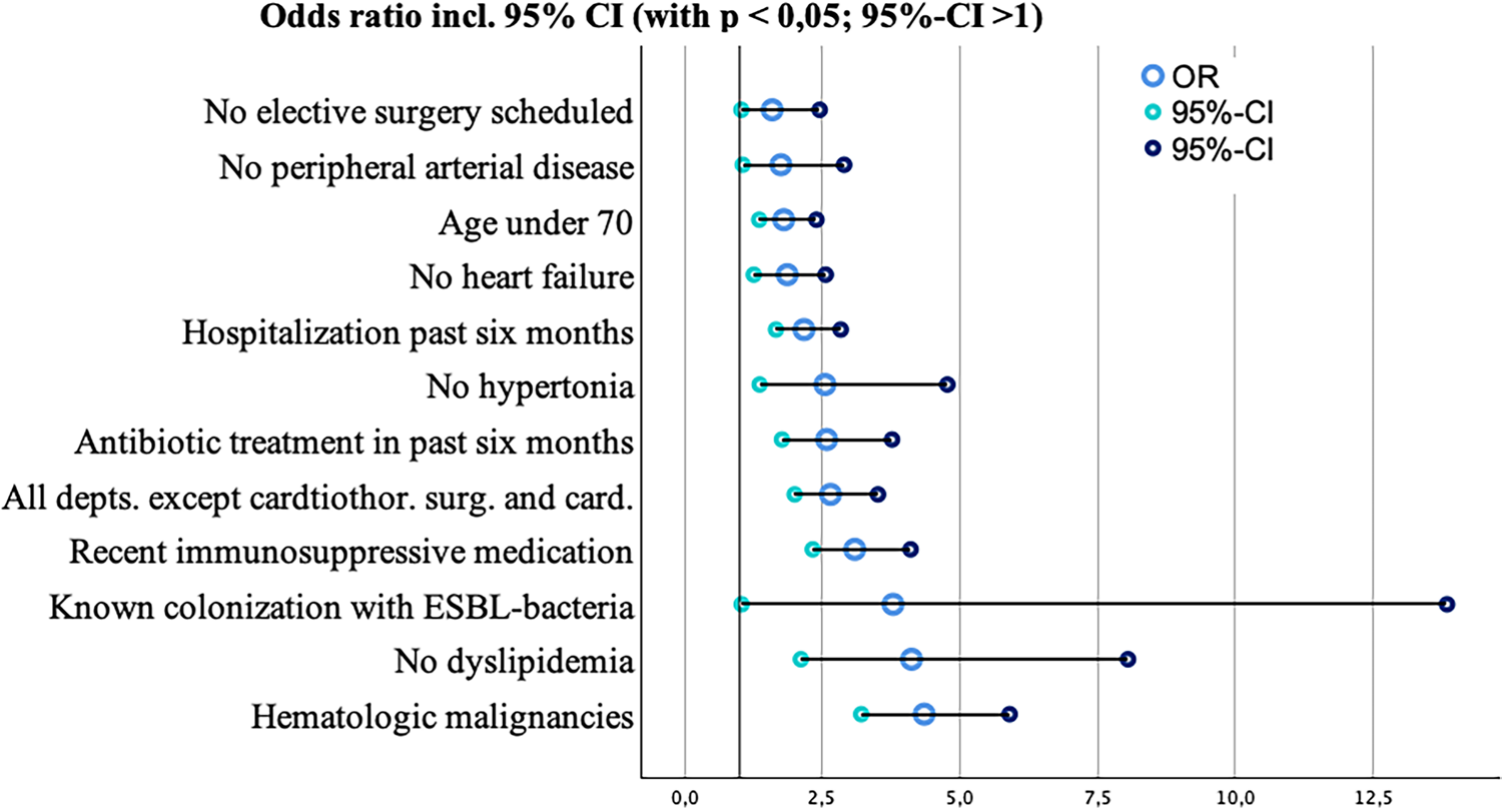
Forest-plot of variables highly correlated with the endpoint (based on univariate regression models). *depts.* departments, *cardiothor. surg. and card.* cardiothoracic surgery and cardiology

Univariate analysis using Pearson's chi-square test and univariate regression revealed several significant (p < 0.05) risk factors for receiving antibiotic treatment during hospitalization (see Supplementary Table 4 and Figure 4) with the most pronounced one being hematologic malignancies.

### Binary logistic regression models

**Fig 5:**
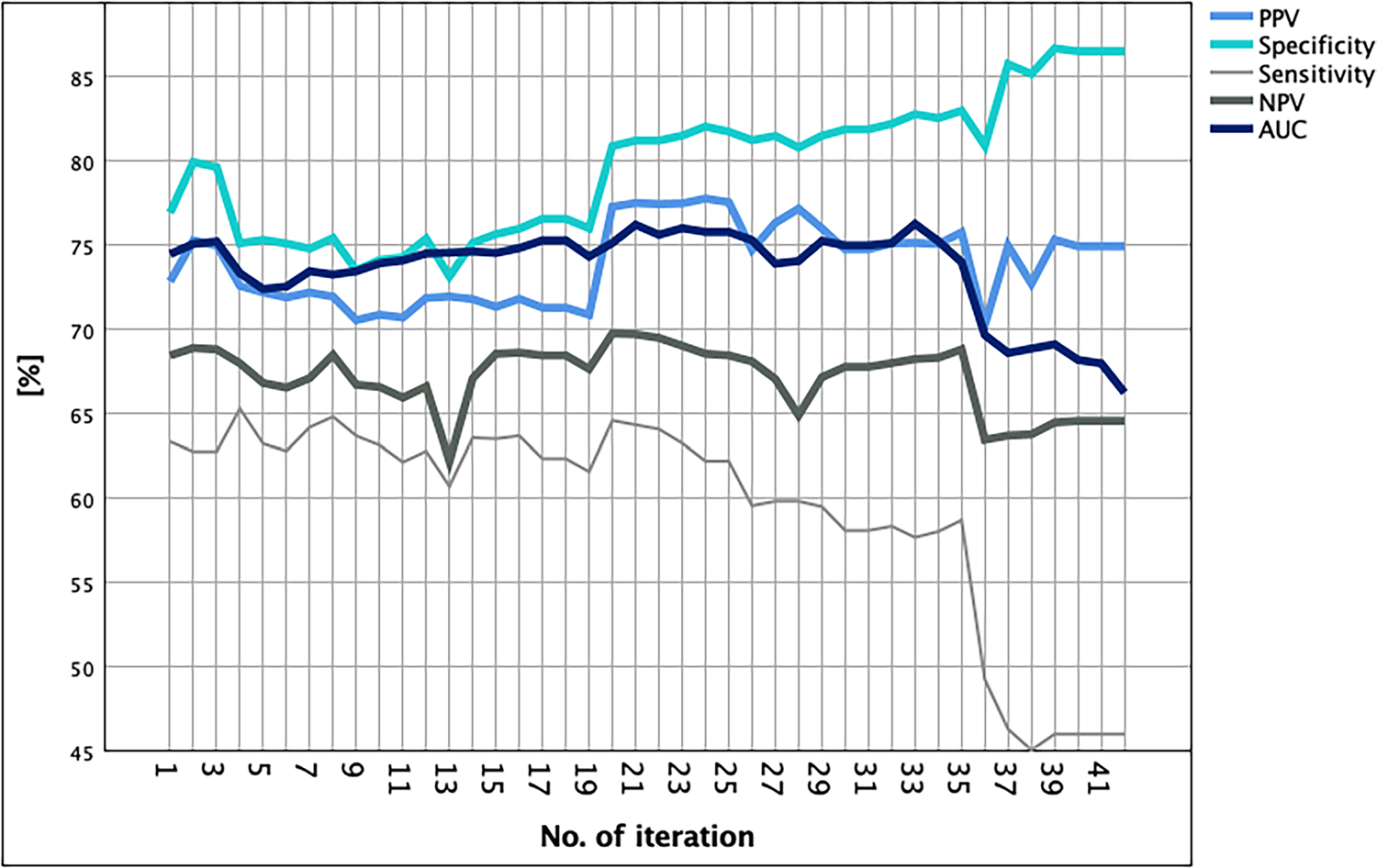
Development of key performance metrics over the course of 41 logistic regression models starting with all available variables (iteration 1)

Logistic regression models were calculated, starting with all defined variables (n = 41, see Supplementary Table 4) and ending with only one variable left (hematologic malignancies), supported by 10-folds cross validation resulting in > 410 calculated models. The development of the key performance indices (sensitivity, specificity, PPV, NPV, AUC) per model is displayed in Figure 5.

The step-wise removal of variables led to significant fluctuations in the metrics but especially for PPV and NPV no major changes comparing first and last iteration were noticeable (see Figure 5). Specificity improved significantly over time, whereas AUC and sensitivity worsened by the number of variables removed from the model. In iteration 36 all metrics simultaneously dropped, so the development of the final model started with the iteration no. 35 balancing the highest metrics with only eight variables left. Iteration 35 yielded a sensitivity of 58.7%, specificity of 83%, PPV of 75.8%, NPV of 68.8% and an AUC of 0.74. Henceforth 14 variations of iteration 35 were calculated trying to further improve model performance and simultaneously reducing the number of included variables. The final logistic regression model contained four variables: (1) hematologic malignancies, (2) recent immunosuppressive medication, (3) hospitalization in the past six months and (4) no elective surgery scheduled. This model yielded a satisfactory model performance while performance improved when calculated on the validation dataset (see Table 3).

**Table 3:** Final regression model performance metrics on the development and validation dataset

	Development dataset (n = 942)	Validation dataset (n = 316)	**∆**
Sensitivity [%]	50.7	50.0	− 0.70%
Specificity [%]	86.5	87.6	1.10%
PPV* [%]	76.9	75.0	− 1.90%
NPV* [%]	66.1	70.1	4.00%
AUC*	0.737	0.748	0.011
Standard error	0.053	0.029	− 0.024
Significance	0.002	< 0.001	− 0.002

*PPV* positive predictive value, *NPV* negative predictive value (NPV), *AUC* area under the receiver operating characteristic curve

### Score development

When calculating the individual variable weights within the score based on the final logistic regression model and according to the three methods for weighting described above, the variable “no elective surgery” resulted in a negative regression, in contrast to its previously positive coefficient (0.46) in the univariate analysis, where it had an odds ratio (OR) of 1.59 (95% CI 1.22–2.06). This resulted in negative weightings for the three remaining variables and a positive weighting for the variable “no elective surgery”. To address this, the model was adjusted by replacing the variable “no elective surgery” with the turned variable “elective surgery planned” as consistent predictor directions were needed for meaningful weight calculations. The weighted score methods assigned higher values to stronger predictors, such as hematological malignancies, which received the highest weight across all methods (see Table 4). The weightings were rounded to one decimal place, with the largest range found in the Schneeweis method (up to 4 points), assigning 5 points for hematological malignancies and 1 point for prior hospitalization, while the Matsushita method showed a smaller range of 2.2 points.

**Table 4:** Results of individual weight calculations per weighting method and variable, compared to reference and maximum points per method

Variables	Ø regression-coefficient	Ø OR	Werfel	Matsushita	Schneeweiss	Unweighted
Immunosuppressive medication	0.71	2.04	2	1.3	2	1
Hematologic malignancies	1.57	4.83	4.4	2.9	5	1
Hospitalization (past 6 months)	0.36	1.43	1	0.7	1	1
Elective surgery planned	0.88	2.42	2.5	1.7	3	1
Max. points per method (∑ of individual weightings)			9.9	6.6	11	4

### Threshold analysis

For each patient case in the development dataset, the theoretically achievable score was calculated using the additive score models by Werfel, Schneeweiss, and Matsushita [10–12]. By comparing these scores with actual outcomes, different model thresholds were simulated to predict whether an endpoint was positive or negative. Across all methods, higher thresholds led to higher specificity and PPV, but lower sensitivity and NPV. For example, Werfel's method showed a specificity of 89% and PPV of 75% at a threshold of 6 points, but sensitivity and NPV remained low at 38% and 61%. Similar results were found for the Matsushita and Schneeweiss methods, with higher thresholds consistently improving specificity but reducing sensitivity. When comparing with the unweighted reference calculation (every variable was assigned weight = 1) a stable predictive performance was noticeable with a specificity of 93% and a PPV of 79% (sensitivity: 30%, NPV: 59%) questioning the need for individual variable weighting. The weighted and unweighted scores were validated with the validation dataset and again showed stable performance metrics: specificity 92%, PPV 76%, sensitivity 37%, NPV 67% (weighted version, threshold 60%) and specificity 94%, PPV 77%, sensitivity 27%, NPV 65% (unweighted version, threshold 75%). For practicality reasons the final score model based on the Werfel method was calculated with integer weightings:

- Hematologic malignancies: 5 points
- Immunosuppressive medication: 2 points
- Hospitalization past 6 months: 1 point
- Elective surgery planned: 2 points

**Fig 6:**
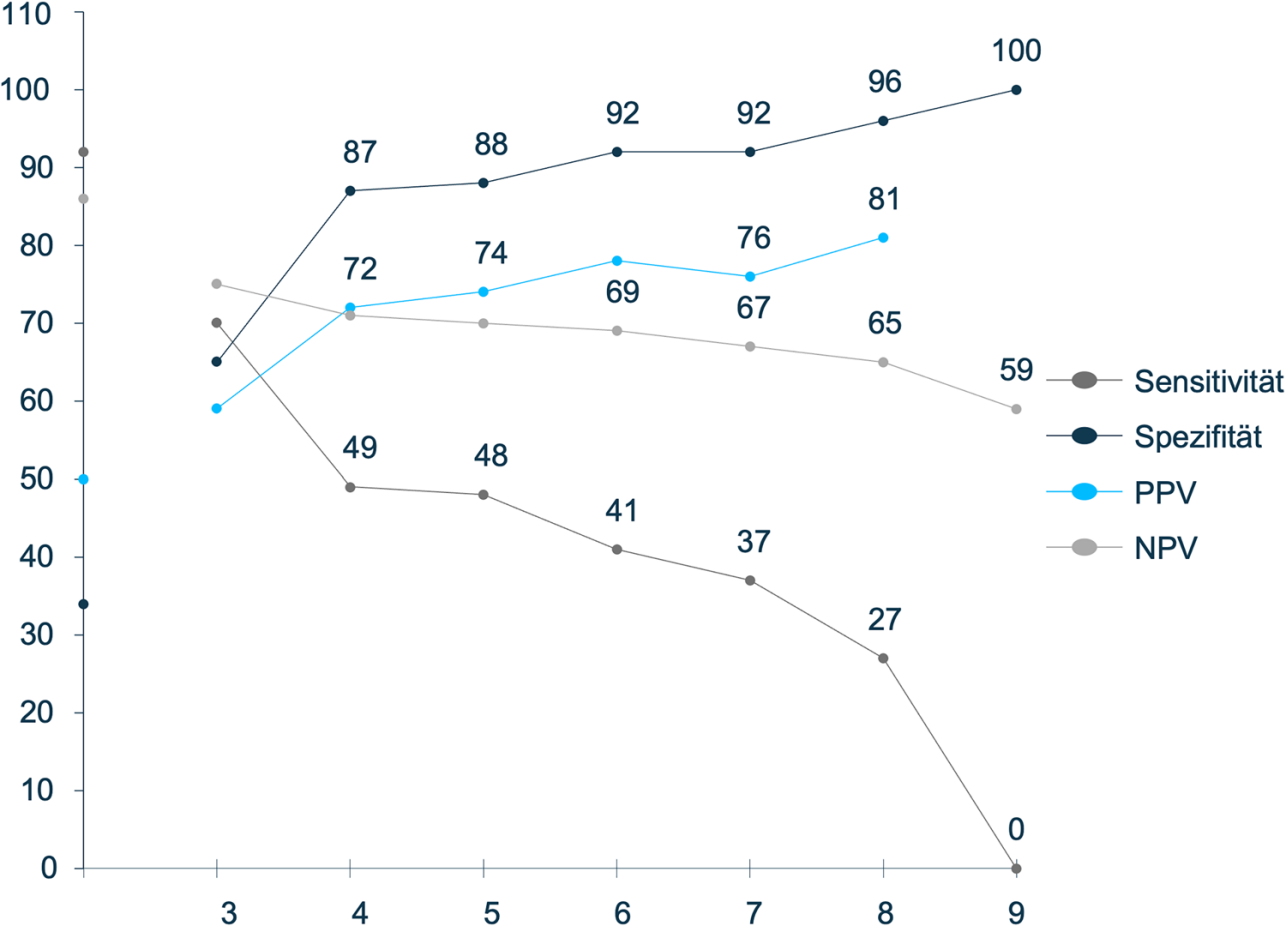
Score performance per threshold using integer weightings based on the Werfel method

Depending on threshold (max. points: 10) the score yielded good performance as shown in Figure 6. Overall, while specificity and PPV were strong, sensitivity and NPV stayed low, limiting their predictive value at higher thresholds. These results indicate that the PILGRIM score is effective at identifying patients unlikely to require antibiotics, as evidenced by its high specificity and PPV.

## Discussion

The PILGRIM score, developed through a comprehensive modeling approach, demonstrated notable accuracy with 92.0% specificity, 76.0% PPV, 37.0% sensitivity, and 67% NPV at a 60% threshold, making it a valuable tool to improve screening procedures for AMS measures and studies. We identified hematologic malignancies, immunosuppressive medication, previous hospitalization, and the planning of elective surgery as key variables. The multinational PILGRIM trial aimed to enroll patients at high risk of requiring subsequent courses of antibiotic treatment following hospital admission. However, less than half of the enrolled patients received antibiotics, despite initial high-risk assessments by the recruiting physician. The robust performance metrics of the PILGRIM score suggest potential for achieving better results in targeted patient inclusion for future AMS-studies and interventions. In AMS studies, sample collection e.g. of stool samples for subsequent microbiome analysis before and after antibiotic therapy can lead to a waste of resources if the patients ultimately do not receive antibiotics. Similarly, AMS interventions could be more effective if clinical AMS teams are able to identify patients at high risk for antibiotic use upon their admission, allowing closer monitoring and timely feedback to the treating physicians when antibiotic therapy is initiated.

Previous studies have identified similar risk factors for infections, yet these have typically been explored individually rather than integrated into a comprehensive predictive model [13–19]. Poran et al. developed a similar logistic regression model, which to our knowledge is the sole publication in this domain to date [8]. Achieving comparable AUC values (0.720–0.750) their cohort was limited to patients from internal medicine departments, did not count repeated admissions but included broader data (e.g., lab results and vital signs). This is in contrast with our model, incorporating surgical patients and considering repeated hospitalizations as major risk factor.

In our model, hematological malignancies, such as leukemia and lymphoma, emerged as highly significant risk factors with the greatest effect size among all variables examined. These conditions disrupt both the innate and adaptive immune systems, thus increasing the risk of severe, life-threatening infections, including those caused by multidrug-resistant organisms. Chemotherapy and newer immunotherapies further exacerbate this vulnerability [20, 21]. Our findings are in contrast with those of Steinberg et al., which indicated that cancer was protective with regard to need for subsequent antibiotic treatment (OR 0,73, 95% KI: 0.52–1.04) [13]. This discrepancy may result from a major difference in patient selection, recruiting only outpatients with documented tobacco use and diagnosed infection rather than hematologic cancer patients as in our study. Similar to our findings, older patients with few comorbidities (e.g., asthma) were less likely to receive antibiotics. Reasons for that may be increased precautions of physicians when prescribing antibiotics due to increased risks of adverse effects or drug interactions in elderly patients or the fact that older patients may present with atypical symptoms of infection. In some cases, end-of-life or palliative care settings may also lead to restricted use of aggressive antibiotic treatments. Matching our findings, immunosuppressive medications have been shown to elevate the risk of infections and thus antibiotic treatment [14, 15]. Previous hospitalizations are also strongly linked to increased infection risk and future antibiotic use, with studies demonstrating that prior hospital stays significantly raise the likelihood of (bloodstream) infections [16]. A mortality analysis by Zeng et al. similarly showed prior hospitalization (90-day timeframe) with a hazard ratio of 2.1 (95% KI: 1.1–3.9, p = 0.02) as a strong risk factor [17]. Elective surgeries appeared protective in our analysis, likely due to routine perioperative antibiotic prophylaxis which was not counted as antibiotic treatment per se in our endpoint-definition, even when administered prolonged. Especially in cardiothoracic procedures which make the greatest proportion of surgical procedures in our cohort, perioperative prophylaxis is standard practice [22, 23]. While not recommended in most cases, antibiotic prophylaxis is often unnecessarily prolonged following the surgery, potentially impacting the incidence of other infections.

Our score’s additive method, which sums variables to generate a total score, offers simplicity and bedside usability. Logistic implementations can be more precise, but the difference in predictive performance is often minimal, suggesting to recommend the additive model for practical use [24, 25]. Similar observations can be made for individual variable weighting. Despite the varying influence of variables in logistic regression models, individualized weighting did not seem to offer a clear advantage in our analysis. For example, meta-analyses of clinical scores like the Wells score including 7,300 patients have shown no significant performance difference between weighted and simplified versions, favoring simplicity for clinical use [26, 27].

The semi-automated approach used for developing the PILGRIM score, including 10-fold cross-validation, was designed to ensure a standardized process and robust results. Although machine-learning approaches hold promise and are widely used to develop predictive models, a meta-analysis of Christodoulou et al. suggests that fully automated approaches not inherently outperform traditional methods as used in this study [10, 28].

The temporal validation method as a form of internal validation used in this analysis can lack generalizability for different cohort characteristics. Steyerberg et al. note that random data splitting can risk imbalances between outcome variables and predictors, so for our study splitting by study inclusion date was chosen to enhance the model's generalizability [29, 30]. Here, a 3:1 data split was selected as a compromise rather than 1:1 or 2:1 to avoid a too small development-dataset. Although internal validation is considered a classic method, it remains valuable, especially if a large validation cohort is available. Alternatively newer methods like bootstrapping are recommended when external validation is not possible [29].

### Limitations

The PILGRIM study cohort was predominantly composed of multimorbid patients mostly from hematological and oncological departments, potentially limiting its generalizability. Further validation in more diverse patient cohorts and prospective settings is needed to confirm the score’s broader applicability and validity. The model did not include laboratory data and vital signs, which could have further improved predictive performance. The moderate sensitivity of the score highlights its limitations in identifying all high-risk patients, emphasizing the need for complementary clinical tools. Compared to the development cohort, the validation cohort included fewer patients receiving antibiotics, however, we do not consider this as a disadvantage as it rather underlines the significance of the validation considering the lower proportion of positive endpoints. Considering the mathematical change of direction of the variable “no elective surgery” this variable may pose a weakness of the score. Nonetheless it was decided to include the variable in the final score due to its significance. During calculations a significant correlation between some of the final variables was noticeable. Multicollinearity can cause unstable standard errors and p-values but does not affect the model’s fit or prediction performance. To address multicollinearity, increasing sample size or creating new variables that account for the relationships between collinear predictors could be effective [31, 32].

## Conclusion

The PILGRIM score is an effective instrument to assess the likelihood for antibiotic treatment in newly hospitalized patients, especially in the hematological and oncological setting. It is particularly beneficial for physicians engaged in Antimicrobial Stewardship (AMS) interventions or conducting clinical studies focusing on AMS, as it enables more precise identification of patients who are at risk for future antibiotic treatment.

## Supplementary Information

Below is the link to the electronic supplementary material.

Supplementary file1 (PDF 2152 KB)

## Data Availability

Data can be made available upon request to the corresponding author.
