# Supplementary material for: Identifying patients at high risk for antibiotic treatment following hospital admission: a predictive score to improve antimicrobial stewardship measures
Source: Infection. 2025 Apr 15;53(5):1941–52. doi: 10.1007/s15010-025-02525-9 (PMC12460503; doi:10.1007/s15010-025-02525-9)
Supplement: Supplementary file 1 — Supplementary file1 (PDF 2152 KB) [file 15010_2025_2525_MOESM1_ESM.pdf]

Supplementary Material

Optimizing Patient Selection for Antimicrobial Stewardship Studies: A Predictive Score

Supplementary Table 1 PILGRIM study inclusion and exclusion criteria

Inclusion criteria	Exclusion criteria
1. Age ≥ 18 years<br>2. Planned treatment or high likelihood of any systemic antibacterial treatment, except prophylaxis with trimethoprim/sulfamethoxazole, within the next 10 days for a duration of ≥ 5 days<br>3. Patients able to provide a stool sample before or within 6 hours of receiving first antibiotic dosage or patients agreeing to provide a rectal swab as first sample if the initial stool sample cannot be provided within 6 hours of receiving first antibiotic dosage<br>**4.** Written informed consent provided prior to inclusion	1. Patients who have received courses of systemic antibacterials for 7 days or more within the past two months<br>2. Patients having received any antibacterial compound other than prophylaxis with trimethoprim/sulfamethoxazole within 14 days prior to study enrolment except first antibiotic dosage within 4 hours prior enrolment<br>3. Patients who participate in clinical trials evaluating antibacterial substances<br>4. Patients with diarrhea at enrolment (≥3 unformed bowel movements within 24h)<br>5. Patients with a stoma (jejunostomy, ileostomy, or colostomy) at time of inclusion<br>6. Patients on enteral (tube fed or PEG) or parenteral nutrition<br>7. Patient with any social or logistical condition which in the opinion of the investigator may interfere with the conduct of the study, such as incapacity to well understand, not willing to collaborate, or cannot easily be contacted after discharge<br>8. Patients exclusively treated as outpatients without prior hospital admission<br>9. Previous participation in this study

Supplementary Table 2 Validation cohort: Basic demographics of patients included with and without antibiotic treatment

	Endpoint positive patients (= received antibiotics) N = 134	Endpoint negative patients (= did not receive antibiotics) N = 182	Correlations (p-value)
Female sex	40.0% (n=53)	40.7% (n=74)	0.843
Age (Median / IQR)	61.0 a / 17.5	65.0 a / 18.0	0.454
BMI (Median / IQR)	27.3 kg/m^2 / 5.9	26.5 kg/m^2 / 6.0	0.469
CCI (Median / IQR)	4.0 / 3.0	4.0 / 3.0	0.492
Past Hospitalization (last 6 months)	50.7% yes (n=68)	31.2% yes (n=58)	**<0.001**
Antibiotics in the past (last 6 months)	18.7% yes (n=25)	8.2% yes (n=15)	**0.006**
Planned elective surgery	38.1% yes (n=51)	48.4% yes (n=88)	0.089
Immunosuppressive drugs	43.3% yes (n=58)	17.0% yes (n=31)	**<0.001**

Supplementary Table 3 Validation cohort: Admitting departments and performed surgeries

Admitting departments	Proportions [%]
Hematological and oncological	30.7
Cardiothoracic surgery	18.7
General surgery	15.5
(General) Internal Medicine	11.1
Cardiology	7.0
Other	16.6
**Type of elective surgery (applicable for patients with planned elective surgery)**	
Cardiac surgery	44.0
Abdominal surgery	37.0
Urology	7.0
ENT*	3.0
Other/Missing	9.0

*Ear nose throat surgery

Supplementary Table 4 Risk factor identification

Patient variables (n= 41) assessed as part of this analysis. Univariate analysis using Pearson's chi-square test and univariate regression revealed several significant (p<0.05) risk factors for receiving antibiotic therapy during hospitalization (highlighted in bold type).

**Variable**	**P-value[1]**	**Phi**	**Regression Coefficient**	**P-value[2]**	**OR**	**95%CI (OR)**
Hematologic malignancies	**0.000**	0.32	1.47	0.000	4.35	3.20-5.90
All departments except cardiothoracic surgery and cardiology	**0.000**	0.20	0.91	0.000	2.48	1.86-3.30
Risk departments (internal medicine)	**0.000**	0.15	0.97	0.000	2.65	2.00-3.51
Recent immunosuppressive medication	**0.000**	0.15	1.13	0.000	3.09	2.33 - 4.10
Antibiotic treatment in past six months	**0.000**	0.17	0.95	0.000	2.58	1.77 - 3.70
Hospitalization in past six months	**0.000**	0.19	0.77	0.000	2.17	1.66 - 2.83
Risk departments (all departments except surgical departments)	**0.000**	0.23	0.72	0.000	2.06	1.51-2.79
No elective surgery scheduled (admission for other reasons than elective surgery)	**0.000**	0.11	0.46	0.000	1.59	1.22 - 2.06
No dyslipidemia	**0.000**	0.15	1.42	0.000	4.12	2.11 - 8.05
Age under 70	**0.000**	0.13	0.59	0.000	1.80	1.35 - 2.39
No heart failure	**0.000**	0.13	0.62	0.000	1.86	1.26 - 2.56
Medication with Proton-Pump-Inhibitors or H2-Blocker	**0.001**	0.10	0.43	0.001	1.53	1.17 - 2.00
No hypertonia	**0.003**	0.10	0.94	0.003	2.55	1.36 - 4.77
No solid tumor	**0.022**	0.08	0.34	0.022	1.41	1.05 - 1.90

No peripheral arterial disease	**0.029**	0.07	0.56	0.030	1.75	1.06 - 2.90
Known colonization with ESBL-bacteria	**0.031**	0.07	1.33	0.044	3.79	1.04 - 13.84
No recent north-Europe or -America travel	**0.035**	0.07	0.46	0.036	1.59	1.03-2.45
Male sex	**0.044**	0.07	0.27	0.044	1.31	1.01 - 1.71
Known (former) ESBL-infection	0.076	0.06	1.73	0.115	5.64	0.66 - 48.43
Allergy against antibiotics	0.092	0.06	0.40	0.093	1.49	0.94 - 2.36
Known colonization with MDRO	0.097	0.05	0.66	0.102	1.93	0.88 - 4.27
Connective tissue diseases	0.102	-0.05	-0.66	0.108	0.52	0.23-1.16
Recent travel in tropical countries	0.129	0.05	0.82	0.139	2.26	0.77-6.67
Intake diagnosis: heart-valve diseases	0.156	-0.05	-0.28	0.157	0.76	0.51-1.12
Diabetes mellitus	0.177	-0.04	-0.28	0.178	0.75	0.49-1.14
History of myocardial infarction	0.272	-0.04	-0.23	0.273	0.79	0.53-2.00
COPD	0.308	0.03	0.35	0.310	1.42	0.72 - 2.77
Underweight (BMI < 18,5)	0.310	0.03	0.44	0.314	1.56	0.66 - 3.68
History of infection with MDRO	0.334	0.03	0.48	0.339	1.61	0.61-4.26
History of infection with VRE	0.339	0.03	0.81	0.352	2.25	0.41-12.32
Cerebrovascular diseases	0.378	-0.03	-0.28	0.379	0.76	0.41-1.40

Travel to countries with endemic ESBL-E- or Carbapenemase-producing bacteria	0.450	0.03	0.18	0.450	1.19	0.76-1.87
Obesity (BMI > 30)	0.451	0.03	0.11	0.451	1.12	0.84 - 1.50
Hepatic insufficiency	0.509	-0.02	-0.30	0.511	0.74	0.30-1.82
Multimorbid (CCI > 5 points)	0.593	-0.02	-0.89	0.593	0.92	0.66-1.27
Known colonization with VRE	0.625	0.02	0.30	0.627	1.35	0.41-4.44
No travel to foreign countries in past 12 months	0.638	-0.02	-0.65	0.638	0.94	0.71-1.23
Atrial fibrillation	0.663	-0.01	-0.16	0.663	0.85	0.41-1.77
Referral from other hospital than current hospital	0.667	-0.02	-0.16	0.667	0.85	0.41-1.76
Moderate to severe chronic kidney disease	0.840	0.01	0.07	0.840	1.07	0.57 - 2.00
Asthma bronchiale	0.850	0.01	0.06	0.850	1.06	0.56 - 2.02

(1: Chi-square; 2: Univariate Regression)
